# Adherence to a Vaccination Schedule in a Simulated HIV Vaccine Efficacy Trial Among Adults in Fishing Communities Around Lake Victoria, Uganda

**DOI:** 10.3390/vaccines13050515

**Published:** 2025-05-13

**Authors:** Sharon Barbara Nabasumba Kalenge, Andrew Abaasa, Teddy Nakaweesi, Victoria Menya Biribawa, Annet Nanvubya, Ali Ssetaala, Juliet Mpendo, Brenda Okech, Matt A. Price, Bernard S. Bagaya, Noah Kiwanuka

**Affiliations:** 1 UVRI-IAVI HIV Vaccine Program, Entebbe P.O. Box 49, Uganda; vbiribawa@iavi.or.ug (V.M.B.); ananvubya@iavi.or.ug (A.N.); assetaala@iavi.or.ug (A.S.); jmpendo@iavi.or.ug (J.M.); bokech@iavi.or.ug (B.O.); 2 MRC/UVRI and LSHTM Uganda Research Unit, Entebbe P.O. Box 49, Uganda; andrew.abaasa@mrcuganda.org; 3 Department of Infectious Disease Epidemiology, London School of Hygiene & Tropical Medicine (LSHTM), London WC1E 7HT, UK; 4 Thermofischer Scientific, Bend, OR 97701, USA; teddyug@gmail.com; 5 Department of Epidemiology and Biostatistics, University of California at San Francisco, San Francisco, CA 94122, USA; mattalexprice@gmail.com; 6 Department of Immunology and Molecular Biology, School of Biomedical Sciences, College of Health Sciences, Makerere University, Kampala P.O. Box 7062, Uganda; bagayabs@yahoo.com; 7 Department of Epidemiology and Biostatistics, School of Public Health, College of Health Sciences, Makerere University, Kampala P.O. Box 7062, Uganda; nkiwanuka@gmail.com

**Keywords:** simulated, vaccine, efficacy, predictors, adherence, schedule, adults, fishing, community, Uganda

## Abstract

Background/Objectives: Fishing communities (FCs) around Lake Victoria have been identified as suitable for future HIV vaccine efficacy trials due to their high HIV incidence rates. To inform trial design and implementation, we evaluated adherence to vaccination schedules and study retention in a simulated HIV vaccine efficacy trial (SiVET) among adults from two fishing communities in Uganda. Methods: A 12-month prospective cohort study enrolled 250 HIV seronegative adults, aged 18–49 years, from one island and one mainland FC. The hepatitis B vaccine was administered at months 0, 1, and 6 to simulate an HIV vaccine regimen. Those testing HIV positive or pregnant were referred for care. Socio-demographic, behavioral, and clinical data were collected at baseline, 6, and 12 months. Poisson regression models with robust standard errors were used to identify factors associated with vaccination completion and retention. Results: Participants’ age ranged between 25–34 years, with a mean age of 27.6 years (SD = 6.4), and 68% of participants were from the mainland and 22% from the island. The overall vaccination completion rate was 86.5 per 100 person-years of observation (PYO), and was similar between mainland (86.8/100 PYO) and island dwellers (85.6/100 PYO). Male participants were likelier to complete all vaccinations [aRR = 1.1 (95% CI 1.0–1.2)]. Having received a secondary education or higher was also associated with higher vaccination completion compared to the rates for those with primary or no formal education [aRR = 1.1; 95% CI: 1.0–1.2]. Notably, participants who reported illicit drug use [aRR = 1.3; 95% CI: 1.2–1.4] and those engaged in paid sex [aRR = 1.2; 95% CI: 1.1–1.4] were more likely to complete all study visits. Conclusions: Adherence to vaccination schedules was high and consistent between mainland and island populations. These findings confirm that fishing communities are well-suited for future HIV vaccine efficacy trials. Predictors of adherence include male sex, secondary education, illicit drug use, and involvement in paid sex. High adherence rates underscore the feasibility of conducting such trials in this population.

## 1. Introduction

Despite the availability of efficient prevention measures like antiretroviral therapy (ART) and medical male circumcision, the HIV/AIDS pandemic remains a major global health concern, with disproportionately high rates of transmission in low- and middle-income countries (LMICs) [1,2,3]. A total of 39.9 million were estimated to be HIV positive, 1.3 million new cases were reported, and 630,000 deaths from HIV-related causes were recorded worldwide by the end of 2023 [4]. The HIV/AIDS prevention interventions necessitate regular adherence, which is frequently impeded by financial limitations, logistical obstacles, and stigma, especially in environments where social vulnerability is high and care-seeking behaviors are few [2,5]. Furthermore, a recent study regarding how international funding cuts affect the worldwide response to HIV has brought attention to the magnitude of the imminent new infections and HIV related deaths. The study showed that by 2030, there may be an additional 4.43 to 10.75 million new HIV infections, including up to 880,000 in children. Between 770,000 and 2.93 million additional people may die from HIV-related causes during that time, with up to 120,000 of those fatalities occurring in children, especially in Sub-Saharan Africa [6]. An effective HIV vaccine remains the most promising solution for achieving sustained control of the HIV/AIDS epidemic [7,8]. Unlike behavioral or biomedical remedies that require continual adherence, vaccines offer long-term protection, removing the need for people to constantly prioritize HIV prevention in the face of conflicting goals and budget constraints [1,7,8]. This would be especially beneficial for populations at high risk of HIV infection who may not prioritize prevention measures due to costs, time constraints, or challenges in accessing healthcare systems [9].

The launch of the COVID-19 vaccines has highlighted another critical barrier: vaccine hesitancy. Alarming levels of vaccination refusal have been observed worldwide, including in Uganda, due to anxieties about vaccine safety, efficacy, and trust in healthcare systems [1,10,11,12]. Addressing these issues early through community engagement is essential to ensure that at-risk populations are adequately prepared for [10] and willing to participate in the development and testing of HIV vaccines. Currently, several HIV vaccine candidates are advancing through early clinical trials, with a few progressing to Phase IIb and III efficacy trials [13,14]; thus, understanding the roots of mistrust and scepticism will be key to fostering acceptance and participation in future HIV vaccine trials.

Fishing communities around Lake Victoria in Uganda have emerged as potential target populations for HIV vaccine trials due to their exceptionally high HIV incidence rates [15,16,17,18]. Observational cohorts have shown that these populations exhibit favorable traits for long-term research, such as high retention rates and a willingness to take part in clinical trials [15,18,19,20].

Despite the suitability of fishing communities, the success of any HIV vaccine efficacy trial hinges on participants’ adherence to vaccination schedules, consistent follow-up, and accurate reporting of reactogenicity and adverse events [21]. To understand this, we conducted a prospective simulated HIV vaccine efficacy trial (SiVET) among adults from two fishing communities near Lake Victoria, Uganda. Using the hepatitis B vaccine as a proxy for an experimental HIV vaccine, we measured adherence to the vaccination schedules. We identified socio-demographic and clinical predictors of adherence and retention.

## 2. Materials and Methods

Study design and setting: This was a 12-month prospective cohort study mimicking a vaccine efficacy trial, using the hepatitis B vaccine as a proxy for an HIV vaccine. The study was conducted among two fishing communities on Lake Victoria, Uganda. The selection of the two study communities was based on the researchers’ previous experience in these fishing communities [19,22]. One community was located on the mainland and the other on an island. Fishing communities have been defined as heavily populated areas where livelihoods are directly or indirectly linked to fishing activities [17]. These communities often face limited social amenities and high HIV prevalence and incidence rates [15,16,18,23].

Participant Recruitment and Eligibility: We worked closely with community leadership, peer leaders, and an existing community advisory board to identify potential participants and ensure that recruitment procedures were acceptable. Eligibility criteria included being 18–49 years old, HIV uninfected and negative for hepatitis B surface antigen (participants with positive anti-hepatitis B antibodies were included in the study, as hepatitis B vaccination among these participants is neither harmful nor beneficial. Participants with positive anti-HBs antibodies who were given hepatitis B vaccination were counseled prior), having resided in the fishing community for at least six months, and agreeing to 13–18 months of follow-up. Female participants were required to be willing to use long-term contraception.

Initial HIV testing followed the national algorithm using rapid tests (Alere Determine™ HIV-1/2, Abbott Diagnostics Scarborough Inc., Abbott Park, IL, USA, STATPak Dipstick, Chembio Diagnostic Systems Inc., New York, NY, USA, and Unigold™ HIV, Trintiy Biotech Plc Co., Wicklow, Ireland). Participants identified as HIV-infected were excluded and referred for care. Screening for hepatitis B surface antigen (HBsAg), hepatitis B surface antibody (Anti-HBs), and hepatitis B core antibody (Anti-HBc) were performed, and those positive for HBsAg were excluded and referred for further care. Syphilis testing was conducted using the MacroVue^TM^ RPR card test, Becton, Dickinson, and Company, Franklin Lakes, NJ, USA. A confirmatory test was performed using the ABON Syphilis Ultra Rapid test kit, Abon Biopharm (Hangzhou) Co., Ltd., Hangzhou, China. Antibody titers were obtained on the BD Macrovue reactive samples. Those displaying titers above 1:2 and were positive for ABON TPHA were considered syphilis-positive, were treated, and were allowed to continue their participation. All female participants were tested for pregnancy. This was conducted using the Quick Vue One step hCG Combo test, Quidel Corporation, San Diego, CA, USA on urine. Women found to be pregnant were excluded. Family planning counseling and contraceptives were provided to female participants, with those already using contraception encouraged to continue. Participants also completed an interviewer-administered questionnaire regarding socio-demographics, medical history, and questions from the WHO AUDIT-C survey for alcohol use.

Intervention and follow-up: Participants received 1 mL intramuscular injections of the hepatitis B vaccine at months 0, 1, and 6. Follow-up visits occurred at months 6 and 12. Hepatitis B surface antibody (anti-HBs) titers greater than or equal to 10 mIU/mL were considered protective [24]. All participants who were found to present a hepatitis B infection were referred to Mulago National Hospital for further evaluation. Socio-demographic, behavioral, and clinical data were collected at baseline, 6, and 12 months.

Participants were reimbursed between USD 1.33 to USD 2.66 for time and transport at each visit, as per the research ethics committee recommendation and approval.

### Statistical Methods

Data management: Study data were double-entered and cleaned using MS Access, 2016 (Microsoft Corporation, Redmond, WA, USA).

Data analysis: This analysis aimed to achieve the following:

1. To determine the vaccination completion rate among participants enrolled in a simulated efficacy trial.
2. To determine the factors associated with completing all vaccination visits in a simulated efficacy trial.

Data were analyzed using STATA version 14.0 (StataCorp, College Station, TX, USA). Independent variables included age, sex, site, tribe, education, marital status, religion, occupation, and duration of community stay. These were summarized using means, standard deviations (for age), frequencies, and proportions and were stratified by sex and dependent variable.

The dependent variable was the vaccination completion rate, calculated as the number of participants completing all three vaccinations divided by the total person-years of observation (PYO). The PYO were calculated as the aggregated participants’ time from the date of enrolment to the date of the 12-month visit or date of censoring. Multi-variable Poisson regression models with robust standard errors were used to estimate adjusted rate ratios (aRRs) and 95% confidence intervals (CIs) for the factors associated with the dependent variable. In the bivariable (unadjusted) analysis, factors that attained statistical significance at a *p*-value < 0.2 were considered for multivariable (adjusted) analysis, except for having sex with a new partner(s) in the last three months, which was considered a prior. Adjusted model statistical significance was based on a *p*-value of <0.05.

Ethical and Human Subjects Consideration: The study was approved by the Uganda Virus Research Institute Science and Ethics Committee, reference number GC/127/15/07/439, and Uganda National Council of Science and Technology, reference number HS1850. All participants provided written informed consent before participating in any study procedures.

## 3. Results

### 3.1. Participants’ Sociodemographic Characteristics

**Figure 1:**
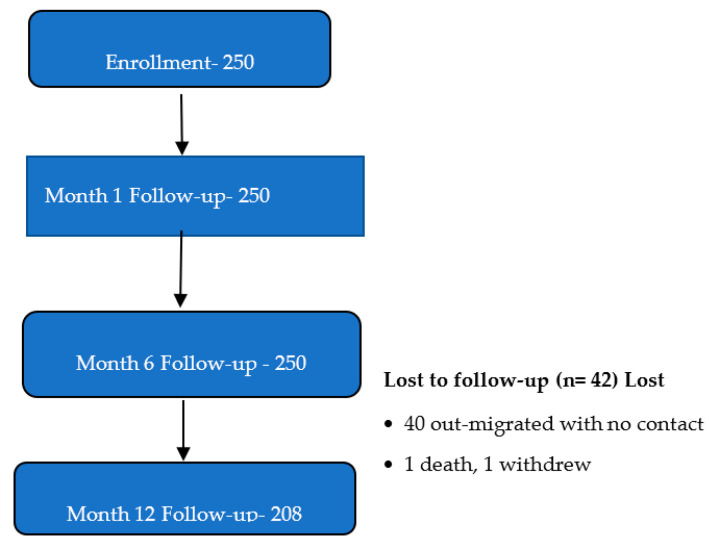
Study flow diagram.

A total of 250 participants were enrolled in the study [see Figure 1], ranging in age from 18 to 49 years, with a mean age of 27.6 years (SD = 6.4). Women were slightly older, on average (mean = 28.6 years, SD = 6.1), compared to men (mean = 27.2 years, SD = 6.5). Nearly half of the participants were between 25 and 34 years old. The majority were from the mainland fishing community [128/187 (68.5%)], male [91/187 (48.7%)], unmarried [102/187 (54.6%)], had attained a secondary-level education [87/187 (46.5%)], and were engaged in fishing-related occupations [86/187 (46%)] [see Table 1].

### 3.2. Completion of the Vaccination Schedule

Overall, 208 participants (83.2%) completed all three vaccination visits within the scheduled timeline. The vaccination visit completion rate was 86.5 per 100 PYO, similar to that for the mainland (86.8/100 PYO) and island dwellers (85.6/100 PYO); see Table 2.

#### Factors Associated with Vaccination Completion

Male participants were more likely to complete all vaccinations compared to females [aRR = 1.1; 95% CI: 1.0–1.2]. Secondary education or higher was associated with higher vaccination completion compared to primary or no formal education [aRR = 1.1; 95% CI: 1.0–1.2]; see Table 3. Notably, participants who reported illicit drug use [aRR = 1.3; 95% CI: 1.2–1.4] and those engaged in paid sex [aRR = 1.2; 95% CI: 1.1–1.4] were more likely to complete all study vaccination visits.

### 3.3. Retention in the Study

The overall study retention rate was 72%, with slightly higher retention observed among males (72%) compared to females (70%). Participants from the mainland community also demonstrated better retention (75%) compared to those from the island (64%). Additionally, younger participants (adolescents and young adults aged 18–24 years) exhibited higher retention rates (74%) compared to that of older adults (71%). Higher retention was also noted among individuals who had attained at least a secondary education (77%), those who were married (75%), and those engaged in non-fishing-related occupations (73%). Furthermore, participants who had resided in the community for more than one year were retained at a higher rate (74%) compared to that for those with shorter durations of residence. See Table 4.

#### Factors Associated with Retention in the Study

Mainland participants were marginally more likely to complete the study compared to island dwellers, although this association did not reach statistical significance [adjusted rate ratio (aRR) = 1.1; 95% confidence interval (CI): 0.9–1.4]. Similarly, participants with a secondary or higher education were as likely to complete all study visits as those with less education [aRR = 1.1; 95% CI: 0.9–1.3].

Participants who had lived in the community for more than one year were 1.2 times more likely to complete all scheduled visits compared to those who had lived there for one year or less, although this difference was not statistically significant [adjusted odds ratio (aOR) = 1.2; 95% CI: 0.9–1.6], as summarized in Table 5.

### 3.4. HIV Incidence

HIV incidence was high among females aged between 25 to 34 years residing in mainland fishing communities [3.5/100 PYAR (95%CI 0.87–13.92)]; see Table 6.

## 4. Discussion

Our study demonstrates high adherence to vaccination schedules and overall study retention among adults in fishing communities. These findings confirm the suitability of fishing communities for future HIV vaccine efficacy trials. Key predictors of adherence included male sex, secondary education, illicit drug use, and involvement in paid sex.

The vaccination visit completion rate in our SiVET was high, with many participants completing all three scheduled vaccinations within the timeline. This rate is comparable to or higher than that reported in similar studies conducted among other key populations, such as female sex workers in Kampala, Uganda [21], participants in the Kenyan SiVET [25], and another among members of FCs around Lake Victoria, Uganda [26]. The high adherence observed in our study underscores the willingness and ability of fishing community members to participate in structured health interventions, even when multiple clinic visits are required.

Men were more likely to complete all vaccination visits compared to women, a finding consistent with that in previous studies [20,27,28]. Negative experiences with healthcare providers, caregiving duties, and gender differences favoring men can make it difficult for women to adhere to clinical trial protocols [28,29,30]. Addressing these barriers through targeted interventions, such as flexible scheduling and supportive counseling, could enhance female participation in future HIV vaccine efficacy trials.

Participants who had attained a secondary education or higher were more likely to complete all vaccinations compared to those with lower educational attainment. Higher education attainment has been linked to enhanced health literacy and greater awareness of preventive measures, leading to better adherence to treatment plans [31]. Programs aimed at increasing educational opportunities for individuals in fishing communities could potentially improve their engagement in HIV prevention efforts.

Participants who reported illicit drug use and those engaged in paid sex demonstrated higher rates of vaccination completion. These findings contrast with those of earlier research suggesting that substance users might be less willing to accept vaccines [32]. However, they align with evidence showing that individuals at high risk of HIV infection often demonstrate a great desire to protect themselves [33]. Participants who reported illicit drug use might have perceived themselves to be at increased risk of hepatitis B infection. Future HIV vaccine efficacy trial campaigns should consider leveraging the heightened awareness and perceived vulnerability among fishing communities to promote vaccine uptake.

Overall study retention was comparable to rates reported in other SiVETs conducted among key populations [20,21,33]. Several factors influenced retention, including geographic location, education level, marital status, occupation, and duration of stay in the community.

Mainland participants exhibited better retention compared to island dwellers, although this difference did not reach statistical significance. Geographic accessibility may play a role here, as mainland residents may find it easier to attend follow-up visits compared to their island counterparts, who must navigate maritime travel challenges.

As observed for vaccination completion, a secondary education or higher was associated with higher retention rates compared to the rates for those with lower educational attainment. Educated individuals may prioritize health-related commitments and understand the importance of continued participation in longitudinal research studies.

Married individuals and those engaged in non-fishing-related occupations showed better study retention. Marriage may provide social stability and support, facilitating adherence to study requirements. Similarly, stable employment outside the fishing sector may reduce mobility and improve consistency in attendance.

Participants who had lived in the community for more than one year were better retained in the study compared to recent arrivals. A longer duration of stay suggests stronger ties to the community and familiarity with local resources, both of which might enhance commitment to research participation and retention.

HIV incidence was notably high among females aged 25–34 years residing in mainland fishing communities, highlighting the urgent need for effective prevention strategies in this population. High HIV incidence rates justify the selection of fishing communities as priority sites for future HIV vaccine efficacy trials.

### Limitations

While our study provides critical insights into the feasibility of conducting HIV vaccine trials in fishing communities, several limitations warrant consideration. First, the relatively short follow-up period (13 months) may not fully capture the long-term retention patterns required for actual HIV vaccine trials lasting up to 3 years. Second, the use of a licensed hepatitis B vaccine instead of an experimental product limits the generalizability of our findings to real-world scenarios involving novel investigational vaccine candidates. Despite these constraints, our results remain informative for planning future HIV vaccine efficacy trials in these communities.

## 5. Conclusions

### Implications for Future Research and Practice

Our findings suggest that fishing communities around Lake Victoria are well-suited for HIV vaccine efficacy trials due to their high adherence to vaccination schedules, fairly good retention, and high HIV incidence rates. To optimize adherence and retention, future trials should incorporate strategies tailored to address specific barriers faced by fishing community subgroups with lower completion rates, such as women, island dwellers, and individuals with limited education. Additionally, leveraging the motivations and behaviors of high-risk groups, such as illicit drug users and those engaged in paid sex, could enhance recruitment and retention efforts.

In conclusion, this SiVET demonstrates the feasibility of conducting HIV vaccine efficacy trials in fishing communities while identifying key predictors of adherence and retention. By addressing identified challenges and building on observed strengths, HIV prevention researchers can design more effective and inclusive HIV vaccine efficacy trials for this vulnerable yet promising population.

## Figures and Tables

**Table 1:** Participants’ baseline characteristics (N = 250).

		Men (*n* = 187)		Women (*n* = 63)		Total (*n* = 250)	
Characteristic		N	%	N	%	N	%
Age (years)	Mean (SD)			Mean (SD)		Mean (SD)	
	27.2 (6.5)			28.6 (6.1)		27.6 (6.4)	
Age Category (years)							
	18–24	73	39.0	17	27.0	90	36.0
	25–34	91	48.7	35	55.6	126	50.4
	≥35	23	12.3	11	17.4	34	13.6
Site							
	Island	59	31.6	21	33.3	80	32.0
	Mainland	128	68.4	42	66.7	170	68.0
Tribe							
	Baganda/Basoga	82	43.9	27	42.9	109	43.6
	Banyankole/Bakiga	36	19.3	10	15.9	46	18.4
	Banyarwanda	16	9.5	6	9.5	*22*	8.8
	Other	53	28.3	20	31.7	73	29.2
Education							
	Primary/None	82	43.9	31	49.2	113	45.2
	Secondary	87	46.5	25	39.7	112	44.8
	More than secondary	18	9.6	7	11.1	25	10.0
Marital Status							
	Not married	102	54.6	22	34.9	124	49.6
	Currently married	85	45.4	41	65.1	126	50.4
Religion							
	Christian	156	83.4	54	85.7	210	84.0
	Muslim	31	16.6	9	14.3	40	16.0
Occupation							
	Fishing-related ^+^	86	46.0	14	22.2	100	40.0
Small scale business		9	4.8	14	22.2	23	9.2
	Services (construction, loading, and off-loading)	11	5.9	5	7.9	16	6.4
	Housewife/student	13	7.0	12	19.1	25	10.0
	Civil service (government employee)	26	13.9	6	9.5	32	12.8
Other (student, farming, shop attendant, unemployed, etc.)		42	22.4	12	19.1	54	21.6
Duration (years) lived in community							
	0–1	37	19.8	7	11.1	44	17.6
	2–4	54	28.9	19	30.2	73	29.2
	5 ^+^	96	51.3	37	58.7	133	53.2

^+^ Fish salting or smoking.

**Table 2:** Rates of vaccination completion by participant baseline characteristics (N = 250, 208 completed trial).

Characteristic	Sub-Category	N/PYO	Rate/100 PYO	95% CI	*p*-Value
Overall		208/240.6	86.5	81.4–90.1	
Sex					
	Female	56/68.9	81.3	70.4–88.6	
	Male	152/171.7	88.5	82.7–92.3	0.579
Age (years)					
	18–24	73/81.2	89.9	81.7–94.9	
	25+	135/159.4	84.7	78.5–89.6	0.681
Site					
	Island	65/75.9	85.6	75.9–91.7	
	Mainland	143/164.7	86.8	80.6–91.0	0.938
Tribe					
	Baganda/Basoga	91/104.4	87.2	79.8–92.5	
	Other	117/136.2	85.9	79.2–90.9	0.903
Education					
	Primary/None	87/105.1	82.8	74.5–88.9	
	Secondary ^+^	121/135.5	89.3	82.6–93.2	0.612
Marital Status					
	Not married	101/119.1	84.8	77.4–90.2	
	Currently married	107/121.5	88.1	80.7–92.4	0.813
Religion					
	Christian	174/202.9	85.7	80.2–89.9	
	Muslim	34/37.7	90.3	75.9–95.8	0.839
Occupation					
	Fishing-related ^+^	76/86.0	88.4	79.9–93.6	
Other (Small-scale business, services, farming, etc.)		132/154.6	85.4	78.7–89.9	0.797
Duration (years) lived in community					
	0–1	35/39.7	88.1	73.9–94.5	
	>1	173/200.9	86.1	80.6–90.2	0.822

^+^ Fish salting or smoking; PYO, person-years of observation; CI, confidence interval.

**Table 3:** Factors associated with completing all vaccination visits (N = 250).

Characteristic	Sub-Category	uRR (95% CI)	*p*-Value	aRR (95% CI)	*p*-Value
Sex					
	Female	1.00		1.00	
	Male	1.09 (0.99–1.19)	0.053	1.09 (1.01–1.20)	0.047
Age (years)					
	18–24	1.00		1.00	
	25+	0.94 (0.87–1.02)	0.156	0.95 (0.87–1.03)	0.215
Site					
	Island	1.00			
	Mainland	1.01 (0.93–1.10)	0.732		
Tribe					
	Baganda/Basoga	1.00			
	Other	0.99 (0.91–1.07)	0.722		
Education					
	Primary/None	1.00		1.00	
	Secondary ^+^	1.08 (0.99–1.17)	0.070	1.10 (1.01–1.19)	0.036
Marital Status					
	Not married	1.00			
	Currently married	1.04 (0.96–1.13)	0.376		
Religion					
	Christian	1.00			
	Muslim	1.05 (0.96–1.16)	0.284		
Occupation					
	Fishing-related ^+^	1.00			
	Other (small-scale business, services, farming, etc.)	0.97 (0.89–1.04)	0.383		
Duration (years) lived in community					
	0–1	1.00			
	>1	0.98 (0.88–1.080	0.673		
Alcohol consumption, last 3 months					
	Never	1.00			
	Sometimes	0.95 (0.88–1.03)	0.256		
Illicit drug use, last 3 months (khat/miraa, marijuana, other)					
	No	1.00		1.00	
	Yes	1.22 (1.13–1.30)	<0.001	1.26 (1.16–1.36)	<0.001
Number of sex partners, last 3 months					
	0–1	1.00		1.00	
	>1	0.91 (0.80–1.04)	0.176	0.88 (0.75–1.02)	0.090
Sex with new partner(s), last 3 months					
	No	1.00		1.00	
	Yes	0.95 (0.87–1.03)	0.211	0.91 (0.83–1.01)	0.082
Condom use with new sex partner(s) (N = 69), last 3 months					
	Not always ^†^	1.00		1.00	
	Always	0.87 (0.75–1.01)	0.057	0.86 (0.76–0.98)	0.029
Received pay for sex, last 3 months					
	No	1.00			
	Yes	0.84 (0.60–1.16)	0.290		
Paid for sex, last 3 months					
	No	1.00		1.00	
	Yes	1.07 (0.97–1.18)	0.180	1.23 (1.06–1.44)	0.008
History of STIs					
	Yes	1.00			
	No	1.12 (0.98–1.28)	0.083		
Experienced an adverse event (AE)					
	Yes	1.00		1.00	
	No	1.24 (1.02–1.49)	0.029	1.15 (0.93–1.41)	0.194

^†^—never, sometimes, and frequently; ^+^—fish salting or smoking; STI—sexually transmitted infection; uRR—unadjusted rate ratio; aRR—adjusted rate ratio; CI—confidence interval.

**Table 4:** Rates of study completion by participants’ baseline characteristics (N = 250).

Characteristic	Sub-Category	Case/PYO	Rate/100 PYO (95% CI)
Overall		161/224.3	71.8 (61.5–83.8)
Sex			
	Female	42/59.7	70.3 (52.0–95.2)
	Male	119/164.6	72.3 (60.4–86.5)
Age (years)			
	18–24	58/78.9	73.5 (56.8–95.1)
	25+	103/145.4	70.9 (58.4–86.0)
Site			
	Island	43/67.2	64.0 (47.5–86.3)
	Mainland	118/157.1	75.1 (62.7–90.0)
Tribe			
	Baganda/Basoga	75/102.8	72.9 (58.2–91.5)
	Other	86/121.5	70.8 (57.3–87.5)
Education			
	Primary/None	61/93.7	65.1 (50.6–83.7)
	Secondary ^+^	100/130.6	76.6 (63.0–93.2)
Marital Status			
	Not married	74/108.1	68.4 (54.5–86.0)
	Currently married	87/116.2	74.9 (60.7–92.4)
Religion			
	Christian	134/188.4	71.1 (60.1–84.3)
	Muslim	27/35.9	75.3 (51.6–100)
Occupation			
	Fishing-related ^+^	53/76.6	69.2 (52.9–90.6)
	Other (small-scale business, services, farming, etc.)	108/147.7	73.1 (60.6–88.3)
Duration (years) lived in community			
	0–1	23/37.5	61.3 (40.7–92.3)
	>1	138/186.8	73.9 (62.5–87.3)

^+^—fish salting or smoking; PYO—person-years of observation; CI—confidence interval.

**Table 5:** Factors associated with study completion (N = 250).

Characteristic	Sub-Category	uRR (95% CI)	*p*-Value	aRR (95% CI)	*p*-Value
Sex					
	Female	1.00		1.00	
	Male	1.03 (0.88–1.21)	0.736	1.02 (0.86–1.21)	0.819
Age (years)					
	18–24	1.00		1.00	
	25+	0.96 (0.84–1.11)	0.619	0.99 (0.86–1.15)	0.943
Site					
	Island	1.00		1.00	
	Mainland	1.18 (0.99–1.40)	0.071	1.13 (0.94–1.36)	0.199
Tribe					
	Baganda/Basoga	1.00			
	Other	0.97 (0.84–1.12)	0.679		
Education					
	Primary/None	1.00		1.00	
	Secondary ^+^	1.18 (1.01–1.37)	0.033	1.12 (0.95–1.32)	0.172
Marital Status					
	Not married	1.00			
	Currently married	1.10 (0.93–1.28)	0.264		
Religion					
	Christian	1.00			
	Muslim	1.06 (0.89–1.26)	0.528		
Occupation					
	Fishing-related ^+^	1.00			
	Other (small-scale business, services, farming, etc.)	1.06 (0.91–1.23)	0.470		
Duration (years) lived in community					
	0–1	1.00		1.00	
	>1	1.21 (0.94–1.54	0.136	1.24 (0.98–1.56)	0.079
Alcohol consumption, last 3 months					
	Never	1.00		1.00	
	Sometimes	0.78 (0.65–0.94)	0.008	0.77 (0.63–0.93)	0.008
Illicit drug use, last 3 months (khat/miraa, marijuana, other)					
	No	1.00			
	Yes	0.80 (0.50–1.30)	0.372		
Number of sex partners, last 3 months					
	0–1	1.00			
	>1	1.02 (0.81–1.28)	0.850		
Sex with new partner(s), last 3 months					
	No	1.00			
	Yes	1.01 (0.85–1.21)	0.881		
Condom use with new sex partner(s) (N = 69), last 3 months					
	Not always ^†^	1.00		1.00	
	Always	1.20 (0.92–1.55)	0.171	1.29 (0.98–1.68)	0.069
Received pay for sex, last 3 months					
	No	1.00			
	Yes	0.88 (0.42–1.83)	0.731		
Paid for sex, last 3 months					
	No	1.00			
	Yes	1.15 (0.88–1.49)	0.314		
History of STIs					
	Yes	1.00			
	No	1.14 (0.76–1.71)	0.521		
Experienced an adverse event (AE)					
	Yes	1.00			
	No	1.91 (0.31–11.94)	0.487		

^†^—never, sometimes, and frequently; ^+^—fish salting or smoking; STI—sexually transmitted infection; uRR—unadjusted rate ratio; aRR—adjusted rate ratio; CI—confidence interval.

**Table 6:** Factors associated with incident HIV infections.

Variable	Category	Number of Cases	Person-Years at Risk (PYAR)	Incidence/100 PYAR	95% CI
Overall		2	198.4	1.01	0.25–4.03
Sex	Female	2	57.4	3.48	0.87–13.92
	Male	0	141.0	0	-
Age group					
	18–24	0	67.9	0	-
	25–34	2	97.6	2.05	0.51–8.19
	35+	0	32.9	0	-
Site					
	Island	0	71.7	0	-
	Mainland	2	126.7	1.58	0.39–6.31

## Data Availability

The dataset used and analyzed during this study is readily available upon reasonable request from the UVRI-IAVI HIV vaccine program.

## Abbreviations

The following abbreviations are used in this manuscript:

AIDS	Acquired Immune Deficiency Syndrome
HIV	Human Immunodeficiency Virus
IAVI	International AIDS Vaccine Initiative
ICMJE	International Committee of Medical Journal Editors
LSHTM	London School of Hygiene and Tropical Medicine
MRC	Medical Research Council
SiVET	Simulated Vaccine Efficacy Trial
UVRI	Uganda Virus Research Institute

## References

[1] The Lancet Public Health (2021). HIV 40: Inequalities fuel pandemics. Lancet Public Health.

[2] Kumah E., Boakye D.S., Boateng R., Agyei E. (2023). Advancing the Global Fight Against HIV/Aids: Strategies, Barriers, and the Road to Eradication. Ann. Glob. Health.

[3] Simon V., Ho D.D., Karim Q.A. (2006). HIV/AIDS epidemiology, pathogenesis, prevention, and treatment. Lancet.

[4] World Health Organisation (2024). HIV and AIDS: Key Facts. https://www.who.int/news-room/fact-sheets/detail/hiv-aids.

[5] Seeley J.A., Allison E.H. (2005). HIV/AIDS in fishing communities: Challenges to delivering antiretroviral therapy to vulnerable groups. AIDS Care-Psychol. Socio-Med. Asp. AIDS/HIV.

[6] Brink d.T., Martin-Hughes R., Bowring A.L., Wulan N., Burke K., Tidhar T., Dalal S., Scott N. (2025). Impact of an international HIV funding crisis on HIV infections and mortality in low-income and middle-income countries: A modelling study. Lancet HIV.

[7] Fauci A.S. (2017). An HIV Vaccine Is Essential for Ending the HIV/AIDS Pandemic. JAMA.

[8] Kaur A., Vaccari M. (2024). Exploring HIV Vaccine Progress in the Pre-Clinical and Clinical Setting: From History to Future Prospects. Viruses.

[9] Smith A.J., Anderson S.-J., Harris K.L., McGillen J.B., Lee E., Garnett G.P., Hallett T.B. (2016). Maximising HIV prevention by balancing the opportunities of today with the promises of tomorrow: A modelling study. Lancet HIV.

[10] Dhama K., Sharun K., Tiwari R., Dhawan M., Bin Emran T., Rabaan A.A., Alhumaid S. (2021). COVID-19 vaccine hesitancy–reasons and solutions to achieve a successful global vaccination campaign to tackle the ongoing pandemic. Hum. Vaccines Immunother..

[11] Unfried K., Priebe J. (2024). Vaccine hesitancy and trust in sub-Saharan Africa. Sci. Rep..

[12] Ouni P.D., Namulondo R., Wanume B., Okia D., Olupot P.O., Nantale R., Matovu J.K., Napyo A., Lubaale Y.A.M., Nshakira N. (2023). COVID-19 vaccine hesitancy among health workers in rural Uganda: A mixed methods study. Vaccine X.

[13] Brett-Major D.M., Crowell T.A., Michael N.L. (2017). Prospecting for an HIV vaccine. Trop. Dis. Travel Med. Vaccines.

[14] Barouch D.H. (2018). A step forward for HIV vaccines. Lancet HIV.

[15] Kiwanuka N., Ssetaala A., Nalutaaya A., Mpendo J., Wambuzi M., Nanvubya A., Sigirenda S., Kitandwe P.K., Nielsen L.E., Balyegisawa A. (2014). High incidence of HIV-1 infection in a general population of fishing communities around Lake Victoria, Uganda. PLoS ONE.

[16] Asiki G., Mpendo J., Abaasa A., Agaba C., Nanvubya A., Nielsen L., Seeley J., Kaleebu P., Grosskurth H., Kamali A. (2011). HIV and syphilis prevalence and associated risk factors among fishing communities of Lake Victoria, Uganda. Sex. Transm. Infect..

[17] Opio A., Muyonga M., Mulumba N. (2013). HIV infection in fishing communities of Lake Victoria basin of Uganda—A cross-sectional sero-behavioral survey. PLoS ONE.

[18] Seeley J., Nakiyingi-Miiro J., Kamali A., Mpendo J., Asiki G., Abaasa A., De Bont J., Nielsen L., Kaleebu P. (2012). High HIV incidence and socio-behavioral risk patterns in fishing communities on the shores of Lake Victoria, Uganda. Sex. Transm. Dis..

[19] Kiwanuka N., Mpendo J., Nalutaaya A., Wambuzi M., Nanvubya A., Kitandwe P.K., Muyanja E., Ssempiira J., Balyegisawa A., Ssetaala A. (2014). An assessment of fishing communities around Lake Victoria, Uganda, as potential populations for future HIV vaccine efficacy studies: An observational cohort study. BMC Public Health.

[20] Bahemuka U.M., Abaasa A., Ruzagira E., Lindan C., Price M.A., Kamali A., Fast P. (2019). Retention of adults from fishing communities in an HIV vaccine preparedness study in Masaka, Uganda. PLoS ONE.

[21] Mayanja Y., Abaasa A., Namale G., Asiki G., Price M.A., Kamali A. (2019). Factors associated with vaccination completion and retention among HIV negative female sex workers enrolled in a simulated vaccine efficacy trial in Kampala, Uganda. BMC Infect. Dis..

[22] Kamali A., Price M.A., Lakhi S., Karita E., Inambao M., Sanders E.J., Anzala O., Latka M.H., Bekker L.-G., Kaleebu P. (2015). Creating an African HIV clinical research and prevention trials network: HIV prevalence, incidence and transmission. PLoS ONE.

[23] Abaasa A., Asiki G., Price M.A., Ruzagira E., Kibengo F., Bahemuka U., Fast P.E., Kamali A. (2016). Comparison of HIV incidence estimated in clinical trial and observational cohort settings in a high risk fishing population in Uganda: Implications for sample size estimates. Vaccine.

[24] World Health Organisation Hepatitis B. https://www.who.int/teams/health-product-policy-and-standards/standards-and-specifications/norms-and-standards/vaccine-standardization/hep-b.

[25] Mutisya E.M., Muturi-Kioi V., Abaasa A., Nyasani D., Kabuti R.W., Lunani L., Kotikot T., Mundia M., Mutua G., Ombati G. (2022). Feasibility of conducting HIV prevention trials among key populations in Nairobi, Kenya. BMC Public Health.

[26] Abaasa A., Nash S., Mayanja Y., Price M., Fast P.E., Kamali A., Kaleebu P., Todd J. (2019). Simulated vaccine efficacy trials to estimate HIV incidence for actual vaccine clinical trials in key populations in Uganda. Vaccine.

[27] Ssendagire S., Ankunda V., Mande S., Ayebazibwe G.K., GeofreySeremba G., Asio D., Atuhurra E.M., Kafeero P., Kitonsa J., Nabukenya S. Adherence to HIV vaccine dose schedule and associated factors among adults enrolled in a HIV vaccine trial in Uganda. Proceedings of the HIVR4P 2024, the 5th HIV Research for Prevention Conference.

[28] Azad A.D., Charles A.G., Ding Q., Trickey A.W., Wren S.M. (2020). The gender gap and healthcare: Associations between gender roles and factors affecting healthcare access in Central Malawi, June–August 2017. Arch. Public Health.

[29] Adjiwanou V., LeGrand T. (2014). Gender inequality and the use of maternal healthcare services in rural sub-Saharan Africa. Health Place.

[30] Ali H.A., Hartner A.-M., Echeverria-Londono S., Roth J., Li X., Abbas K., Portnoy A., Vynnycky E., Woodruff K., Ferguson N.M. (2022). Vaccine equity in low and middle income countries: A systematic review and meta-analysis. Int. J. Equity Health.

[31] Havelka E.M., Sanfilippo J.E., Juneau P.L., Sherman G., Cooper D., Leggio L. (2024). The effect of alcohol, tobacco, and other drug use on vaccine acceptance, uptake, and adherence: A systematic review. Alcohol Alcohol..

[32] Warren E.A., Paterson P., Schulz W.S., Lees S., Eakle R., Stadler J., Larson H.J. (2018). Risk perception and the influence on uptake and use of biomedical prevention interventions for HIV in sub-Saharan Africa: A systematic literature review. PLoS ONE.

[33] Kabarambi A., Kansiime S., Kusemererwa S., Kitonsa J., Kaleebu P., Ruzagira E. (2022). Predictors of Loss to Follow-Up in an HIV Vaccine Preparedness Study in Masaka, Uganda. Int. J. Environ. Res. Public Health.

